# Risk factors for early-onset hyperuricemia and gout following laparoscopic sleeve gastrectomy: a retrospective study

**DOI:** 10.1186/s12893-025-03306-9

**Published:** 2025-11-14

**Authors:** Chenxi Fu, Yitong Han, Jiarun Chen, Junhang Chen, Yan Chen, Xiaoyu Liang, Longhao Sun

**Affiliations:** 1 Department of General Surgery, Tianjin Medical University General Hospital, 154 Anshan Road, Tianjin, 300052 China; 2 Tianjin Key Laboratory of Precise Vascular Reconstruction and Organ Function Repair, Tianjin, 300052 China; 3 Tianjin General Surgery Institute, Tianjin, 300052 China

**Keywords:** Laparoscopic, Sleeve gastrectomy, Hyperuricemia, Gout, Obesity, Risk factors

## Abstract

**Background:**

To investigate the risk factors associated with early-onset hyperuricemia and gout attacks after Laparoscopic Sleeve Gastrectomy (LSG).

**Methods:**

A total of 535 obese patients who underwent LSG at Tianjin Medical University General Hospital between January 2022 and December 2024 were retrospectively enrolled according to predefined inclusion and exclusion criteria.Baseline and 1-month postoperative data—including demographic characteristics, body composition, and laboratory parameters—were collected to assess the incidence and risk factors of postoperative hyperuricemia and goutattacks.

**Results:**

① The incidence of postoperative hyperuricemia was 65.79% (352/535), and the gout attack rate was 4.67% (25/535). The male-to-female ratio of gout patients was 19:6, with 7 patients having a history of gout.② Multivariate analysis revealed that male sex (OR = 7.360), younger age (OR = 0.97), higher preoperative uric acid level (OR = 1.007), lower preoperative creatinine level (OR = 0.934), and higher postoperative creatinine level (OR = 1.069) were independent risk factors for postoperative hyperuricemia. The predictive model demonstrated good performance (AUC = 0.802).③ Multivariate analysis revealed that male sex (OR = 9.434), history of gout (OR = 11.654), larger change amplitude in total body water percentage (OR = 1.193, ΔTBW%), and larger change amplitude in serum uric acid level (OR = 1.018, ΔSUA%) were independent risk factors for postoperative gout attacks. The predictive model demonstrated excellent performance (AUC = 0.895).

**Conclusions:**

The incidence of early hyperuricemia after LSG is high, while the incidence of gout is relatively low. Patient sex, age, preoperativeserum uric acid, preoperative creatinine, and postoperative creatinine are independent risk factors for hyperuricemia after LSG. Patient history of gout, sex, and the amplitude of changes inTBW% and serum uric acid level are independent risk factors for gout attacks after LSG.

## Introduction

 Obesity has reached epidemic proportions worldwide, with severe obesity (BMI ≥ 35 kg/m²) being linked to significantly elevated morbidity and mortality. It is a well-established driver of a wide range of metabolic and cardiovascular comorbidities, among which hyperuricemia and gout represent significant clinical concerns [1].Obesity is a well-established independent risk factor for both hyperuricemia (HUA) and gout [2]. Substantial evidence indicates that weight loss is associated with reduced serum uric acid (SUA) levels and a decreased incidence of gouty arthritis [3, 4]. The 2020 American College of Rheumatology guidelines specifically recommend weight reduction for management of gout in overweight and obese patients [5]. Laparoscopic sleeve gastrectomy (LSG) has emerged as an effective intervention for morbid obesity, producing substantial and sustained weight loss while ameliorating obesity-related comorbidities [6].Paradoxically, despite the long-term benefits of bariatric surgery on SUA levels [7], both clinical observations and growing research indicate a distinct early postoperative phenomenon. A significant rebound in SUA levels typically occurs within the first month after LSG, accompanied by an elevated risk of acute gout flares [8, 9]. This early metabolic shift establishes the immediate postoperative period as a critical high-risk window for HUA exacerbation and gout attacks. However, the precise risk factors and underlying mechanisms driving this response remain insufficiently characterized.Existing literature has predominantly emphasized the long-term urate-lowering effects of bariatric surgery, while the early perioperative phase has received considerably less attention. Consequently, identifying patients at greatest risk during this vulnerable period is essential for developing targeted preventive strategies.Therefore, this retrospective cohort study aims to determine the independent risk factors for early-onset HUA and gout following LSG, with the goal of informing clinical risk stratification and guiding proactive perioperative management.

## Materials and methods

### Research subjects

This was a retrospective observational study. Data were retrospectively collected from patients who underwent LSG at the Department of General Surgery, Tianjin Medical University General Hospital, between January 2022 and December 2024.The inclusion criteria of this study were:① Body mass index (BMI) ≥ 32.5 kg/m² or BMI ≥ 27.5 kg/m² with comorbid type 2 diabetes mellitus (T2DM), in accordance with the Chinese guidelines for bariatric and metabolic surgery [10]② Age 16–65 years;③ Undergoing primary LSG surgery;④ Agreed to participate in follow-up and had complete clinical data.The exclusion criteria of this study were: ① History of other bariatric surgeries;② Presence of severe comorbidities, such as chronic heart failure, myocardial infarction, stroke, malignancy, or other significant gastrointestinal diseases;③ Unstable psychiatric conditions;④ Incomplete clinical data.Patients with a history of hyperuricemia or gout were included regardless of their preoperative use of urate-lowering therapy (ULT), in order to reflect a real-world clinical cohort and observe the natural postoperative course of serum uric acid after medication withdrawal. However, all ULTs were discontinued immediately after surgery as per our institutional protocol.

### Surgical technique

All procedures were performed by the same surgical team. The five-port laparoscopic technique was employed. The surgical steps were as follows: The greater curvature and fundus of the stomach were fully mobilized.A 38-Fr orogastric bougie was inserted and positioned along the lesser curvature.Using a linear stapler, gastric transection commenced 4–6 cm proximal to the pylorus.Transection continued cephalad along the bougie towards the angle of His.The resection line terminated approximately 1.5 cm from the angle of His.The gastric fundus and greater curvature were completely resected, creating a tubular gastric sleeve with an estimated volume of 60–80 mL.The bougie was then removed.The staple line was reinforced with a continuous seromuscular imbricating suture.

### General conditions and clinical data

“Data from 535 patients were retrospectively analyzed. Patient demographics were recorded, including sex, age, BMI, systolic blood pressure (SBP), diastolic blood pressure (DBP), operative time, and history of gout. Body mass index (BMI) was calculated and recorded using the formula: BMI (kg/m²) = weight (kg)/[height (m)]².

### Laboratory biochemical indexes

All subjects were patients with morbid obesity. Data were collected on the day before surgery and at 1 month postoperatively. The collected data included:① Biochemical parameters: fasting blood glucose (FBG), urea, creatinine(CR), SUA, total cholesterol (TC), and triglycerides (TG).② Body composition parameters: Total Body Water percentage (TBW%), fat-free mass༈FFM), and body fat percentage༈BFP).Body composition was assessed using bioelectrical impedance analysis (BIA). While BIA is a practical and non-invasive method, its accuracy can be influenced by hydration status, which may be altered in the postoperative period. This limitation should be considered when interpreting the results. Patients were required to adhere to the following pre-measurement conditions: Be in a fasted state in the morning.Refrain from exercise for 8 h prior to measurement.Abstain from alcohol consumption for 24 h prior to measurement.Maintain euhydration (normal fluid balance).Be in a rested state.Wear light clothing.Stand barefoot on the device platform.Hold the hand electrodes to perform the body composition measurement.Hyperuricemia was diagnosed according to standard criteria [11], defined as two separate fasting SUA levels exceeding 420 µmol/L (measured on different days) under normal purine dietary conditions. Although the perioperative diet is markedly altered, this definition was retained for diagnostic consistency with established guidelines.Gout was diagnosed by rheumatologists in accordance with the American College of Rheumatology (ACR) diagnostic criteria for gout [12].A history of gout was defined as a patient-reported diagnosis of gout documented in the medical record prior to surgery, which was further verified by a rheumatologist according to ACR criteria where possible.

### Statistical analysis

Statistical analyses were performed using SPSS software (Version 27.0, IBM Corp.). Categorical variables were expressed as frequencies and compared between groups using the χ² test or Fisher’s exact test, as appropriate. Continuous variables were described as median (interquartile range, IQR) and compared between groups using the Mann-Whitney U test for non-parametric data; within-group comparisons of pre- and postoperative measurements were performed using the Wilcoxon signed-rank test. Both univariate and multivariable analyses were conducted using binary logistic regression models to identify independent risk factors for HUA and gout. Results are presented as odds ratios (OR) with corresponding 95% confidence intervals (CI). Predictive/diagnostic performance was evaluated by plotting receiver operating characteristic (ROC) curves and calculating the area under the curve (AUC). All statistical tests were two-sided, and a p-value < 0.05 was considered statistically significant.

## Results

### Patient baseline and postoperative characteristics

A total of 535 patients who underwent LSG were included in this final analysis, comprising 160 males (29.9%) and 375 females (70.1%). The baseline and 1-month postoperative characteristics of the entire cohort are summarized in Table 1. As expected, significant reductions were observed in BMI, TBW%, BFP, FPG, urea, and TG levels one month after surgery (all *p* < 0.01). Conversely, SUA and CR levels exhibited a significant increase postoperatively (*p* < 0.01). The prevalence of HUA increased from 55.89% (299/535) preoperatively to 65.79% (352/535) at one month postoperatively. Twenty-five patients (4.67%) experienced acute gout attacks during the follow-up period; among these, 7 had a pre-existing history of gout.

**Table 1:** Baseline and postoperative characteristics of the study cohort (*N* = 535)

	Baseline	Postoperative (1-month)	*P*-value
BMI (kg/m²)	41.4(37.56,45.51)	36.2(32.84,40.0949)	<0.01
TBW%(%)	41.1(37.81,45.34)	38(34.32,42.35)	<0.01
FFM (kg)	52.8(48.19,58.91)	52.48(47.24,57.7)	<0.01
BFP(%)	46.6(43.1,50.8)	45.6(41.2,50.4)	<0.01
FPG (mmol/L)	5.32(4.79,6.25)	5(4.58,5.7)	<0.01
UREA (mmol/L)	4.3(3.6,5)	3.1(2.5,3.8)	<0.01
CR (µmol/l)	56(49,64)	59(52,70)	<0.01
SUA (µmol/l)	431(379,513)	487(397,592)	<0.01
TC (mmol/L)	4.69(4.18,5.32)	4.65(4.03,5.35)	0.113
TG (mmol/L)	1.89(1.36,2.61)	1.65(1.37,2.03)	<0.01

*BMI* body mass index, *TBW%* total body water percentage, *FFM* fat-free mass, *BFP* body fat percentage, *FPG* fasting plasma glucose, *UREA* blood urea nitrogen, *CR* creatinine, *SUA* serum uric acid, *TC* total cholesterol, *TG* triglycerides

### Factors associated with postoperative hyperuricemia

#### Univariate analysis

Patients developing postoperative HUA were significantly younger (median age: 31.21 years vs. 34.45 years, *P* < 0.001) and had higher rates of the following factors compared to the normouricemic group (*P* < 0.05 for all): Male sex, higher preoperative and postoperative BMI, longer operative time, higher preoperative SBP, higher preoperative and postoperative TBW%, higher postoperative FFM, higher preoperative SUA, higher preoperative and postoperative Cr. No significant associations (*P* > 0.05) were found between postoperative HUA and: preoperative FFM, preoperative or postoperative BFP%, preoperative or postoperative FBG, urea, TC or TG, or history of gout (*P* = 0.983). Detailed results are presented in Table 2.

**Table 2:** Univariate analysis of factors associated with postoperative HUA

Variable	Normal Postop	Postop HUA	*p*-Value
	(*n* = 183)	(*n* = 352)	
Male/Female	17/166	143/209	<0.001
History of Gout	11/172	21/331	0.983
Age(years)	34.45(28.22,40)	31.21(26,37)	<0.001
Preop. BMI (kg/m2)	40.45(35.85,44.75)	41.67(38.82,45.99)	0.001
Postop. BMI(kg/m2)	34.56(31.18,38.68)	36.55(33.54,40.63)	<0.001
Operative time(min)	65(50,85)	70(60,85)	0.006
SBP (mmHg)	132(125,139)	136(126,145)	0.029
DBP (mmHg)	90(81,100)	91(82,101.75)	0.253
Preop. TBW%(%)	40.35(37.09,43.9)	41.69(38.08,46.7)	0.002
Postop. TBW%(%)	37.33(34.3,41)	38.45(34.5,43.38)	0.029
Preop. FFM(kg)	52.65(48.43,57.6)	52.98(48.12,60.53)	0.230
Postop. FFM(kg)	51.28(46.8,56)	53.18(48.09,58.95)	0.013
Preop. BFP(%)	46.4(43.3,50.5)	46.9(42.9,50.8)	0.923
Postop. BFP(%)	45.7(41.4,49.9)	45.5(41.1,50.6)	0.838
Preop. FPG (mmol/l)	5.4(4.82,6.39)	5.29(4.78,6.23)	0.359
Postop. FPG (mmol/l)	5(4.7,5.63)	5(4.5,5.7)	0.278
Preop. UREA (mmol/l)	4.3(3.8,5)	4.2(3.6,5)	0.254
Postop. UREA(mmol/l)	3.2(2.6,3.9)	3.1(2.4,3.8)	0.183
Preop. CR (µmol/l)	54(48,61)	57(50,65)	0.010
Postop. CR (µmol/l)	54(49,62)	62.5(54.25,74)	<0.001
SUA (µmol/l)	396(353,443)	455.5(401,564.5)	<0.001
Preop. TC (mmol/l)	4.69(4.17,5.32)	4.695(4.19,5.33)	0.975
Postop. TC (mmol/l)	4.77(4.18,5.44)	4.59(3.99,5.33)	0.124
Preop. TG (mmol/l)	1.88(1.33,2.57)	1.895(1.38,2.65)	0.538
Postop. TG (mmol/l)	1.61(1.33,1.96)	1.66(1.38,2.04)	0.472

*Preop*. preoperative, *Postop*. postoperative, *BMI* body mass index, *SBP* systolic blood pressure, *DBP* diastolic blood pressure, *TBW%* total body water percentage, *FFM* fat-free mass, *BFP *body fat percentage, *FPG* fasting plasma glucose, *UREA* blood urea nitrogen, *CR* creatinine, *UA* uric acid, *TC* total cholesterol, *TG* triglycerides, *SUA* serum uric acid

#### Multivariate analysis

After adjusting for confounders using logistic regression models, sex, age, preoperative CR, postoperative CR, and preoperative SUA were identified as independent predictors of postoperativeHUA (*P* < 0.05). Male sex was the strongest risk factor (β = 1.996, *P* < 0.001), with males having 7.36 times higher risk than females (95% CI: 3.384–16.005). Each 1-µmol/L increase in preoperative SUA elevated risk by 0.7% (OR = 1.007, 95% CI: 1.004–1.009, *P* < 0.001). Each 1-µmol/L increase in postoperative CR raised risk by 6.9% (OR = 1.069, 95% CI: 1.044–1.094, *P* < 0.001). Age showed a protective effect: each 1-year increment decreased risk by 3% (OR = 0.97, 95% CI: 0.944–0.998, *P* = 0.034). Higher preoperative CR levels paradoxically reduced risk (OR = 0.934, 95% CI: 0.910–0.959, *P* < 0.001). Results are summarized in Table 3.

**Table 3:** Multivariate analysis of factors associated with postoperative HUA

Variable	B	Std.Error	Wald	*p*-Value	OR	95% CI
Gender	1.996	0.396	25.358	<0.001	7.360	3.384–16.005
Age	−0.03	0.014	4.512	0.034	0.97	0.944–0.998
CR	−0.068	0.014	25.074	<0.001	0.934	0.91–0.959
Postop CR	0.067	0.012	30.955	<0.001	1.069	1.044–1.094
SUA	0.007	0.001	27.518	<0.001	1.007	1.004–1.009

### Factors associated with postoperative gout flares

#### Univariate analysis

Patients experiencing postoperative gout attacks had significantly: higher proportion of males (76.0% vs. 27.7%, *P* < 0.001), higher rate of pre-existing gout history (28.0% vs. 4.9%, *P* < 0.001), higher preoperative BMI (44.50 vs. 41.12 kg/m², *P* = 0.003), greater postoperative reduction in ΔTBW% (ΔTBW%: −11.67% vs. −8.07%, *P* < 0.001), larger increase in SUA (ΔSUA%: +31.38% vs. +11.67%, *P* = 0.001), higher postoperative SUA levels (589 vs. 482 µmol/L, *P* < 0.001), and higher postoperative sCr (68 vs. 59 µmol/L, *P* = 0.025). No significant differences (*P* > 0.05) were observed in other postoperative metabolic indicators (e.g., FBG, urea, TC, TG). Detailed results are shown in Table 4.

**Table 4:** Univariate analysis of factors associated with postoperative gout flares

Variable	Normal Postop	Postop Gout	*p*-Value
	(*n* = 510)	(*n* = 25)	
Male/Female	141/369	19/6	<0.001
History of Gout	25/485	7/18	<0.001
Age(years)	32.03(26,38)	33(26.49,37.26)	0.983
BMI (kg/m2)	41.115(37.22,45.49)	44.50(41.70,50.89)	0.003
Postop BMI(kg/m2)	36.15(32.73,39.99)	37.91(34.665,42.85)	0.075
Surgical Tim (min)	70(60,85)	65(60,85)	0.911
SBP(mmHg)	135(125,142)	137(125.5,158.5)	0.322
DBP(mmHg)	90(81,101)	92(84,104)	0.245
TBW%(%)	40.94(37.67,45.2)	41.7(39.33,47.05)	0.330
Postop TBW%(%)	38.05(34.3,42.35)	36.18(34.5,42.05)	0.610
ΔTBW%	8.07(5.18, 10.94)	11.67 (10.05, 14.65)	<0.001
FFM(kg)	52.68(47.91,58.8)	53.42(49.72,63.3)	0.374
Postop FFM(kg)	52.37(47.08,57.65)	54.14(49.86,58.99)	0.176
BFP(%)	46.65(43.08,50.8)	46.5(43.05,51.2)	0.611
Postop BFP(%)	45.55(41.18,50.4)	46.7(41.55,50.8)	0.665
FPG(mmol/l)	5.31(4.79,6.24)	5.74(4.83,6.67)	0.441
Postop FPG(mmol/l)	5(4.57,5.7)	5.1(4.61,6.25)	0.511
UREA (mmol/l)	4.25(3.6,5)	4.6(3.7,5.4)	0.339
Postop UREA(mmol/l)	3.1(2.4,3.8)	3.1(2.6,3.75)	0.976
CR (µmol/l)	56(49,64)	56(52,69.5)	0.326
Postop CR(µmol/l)	59(51,69.25)	68(59,77)	0.025
SUA µmol/l	431(382.5,515.25)	393(353.5,513)	0.326
Postop SUA(µmol/l)	482(389.75,586.25)	589(479.5,751.5)	<0.001
ΔSUA %	11.67(10.05, 14.65)	31.38(17.48, 67.42)	0.001
TC (mmol/l)	4.71(4.19,5.36)	4.45(4.1,4.97)	0.228
Postop TC (mmol/l)	4.66(4.03,5.3725)	4.39(4.005,5.01)	0.393
TG (mmol/l)	1.89(1.36,2.6)	2.04(1.52,3.14)	0.211
Postop TG (mmol/l)	1.65(1.36,2.02)	1.62(1.4,2.37)	0.487

*ΔTBW%* Total Body Water Percentage, *ΔSUA%* Serum Uric Acid Change Percentage

#### Multivariate analysis

Four variables were confirmed as independent predictors of postoperative gout attacks after adjustment (*P* < 0.05): male sex (OR = 9.434, 95% CI: 2.910–30.581, *P* < 0.001), history of gout (OR = 11.645, 95% CI: 3.543–38.273, *P* < 0.001), ΔTBW% (OR = 1.193 per 1% increase, 95% CI: 1.071–1.329, *P* = 0.001), and ΔSUA% (OR = 1.018 per 1% increase, 95% CI: 1.004–1.031, *P* = 0.010). Results are summarized in Table 5.

**Table 5:** Multivariate analysis of factors associated with postoperative gout flares

Variable	B	Std.Error	Wald	*p*-Value	OR	95% CI
Gender	2.244	0.600	13.989	<0.001	9.434	2.910–30.581.910.581
History of Gout	2.455	0.607	16.353	<0.001	11.645	3.543–38.273
ΔTBW%	0.176	0.055	10.270	0.001	1.193	1.071–1.329
ΔSUA%	0.018	0.007	6.565	0.010	1.018	1.004–1.031

### ROC curve analysis

Postoperative HUA (Table 6): ROC analysis demonstrated that the integrated prediction model for postoperative hyperuricemia significantly outperformed individual predictors (AUC = 0.802 vs. ≤0.713). Among single predictors: Preoperative SUA (AUC = 0.713) and postoperative CR (AUC = 0.677) showed moderate predictive value.At the optimal cut-off of 425.5 µmol/L for preoperative SUA, balanced sensitivity (64.2%) and specificity (68.9%) were achieved.Male sex exhibited high specificity (90.7%) but limited clinical utility due to low sensitivity (40.6%).When analyzed in the direction of risk (i.e., younger age, represented as Inverse Age), age demonstrated modest predictive value (AUC = 0.598).

**Table 6:** ROC curve analysis for postoperative HUA prediction

Factor	AUC	*p*-Value	95% CI	Cutoff	Youden Index	Sensitivit(%)	Specificity(%)
Gender	0.657	<0.001	(0.61, 0.703)	0.5*	0.313	40.6	90.7
Age*	0.598	<0.001	(0.548, 0.648)	0.662	0.158	55.7	60.1
CR	0.568	0.01	(0.517, 0.618)	59.5	0.162	43.5	72.7
Postop CR	0.677	<0.001	(0.631, 0.723)	56.5	0.291	69	60.1
SUA	0.713	<0.001	(0.668, 0.757)	425.5	0.331	64.2	68.9
Integrated Model	0.802	<0.001	(0.765, 0.838)	0.623	0.477	66.9	77.6

*Cutoff 0.5 for binary variables (Male = 1, Female = 0)*,Age*:Inverse Age

**Fig. 1:**
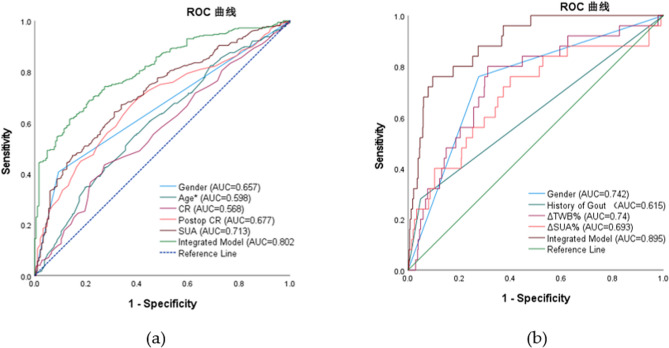
Receiver operating characteristic (ROC) curves for predictive models of (**a**) postoperative hyperuricemia and (**b**) postoperative gout following LSG. The integrated models showed superior predictive performance compared with individual indicators (AUC: 0.802 and 0.895, respectively)

Postoperative Gout Attacks (Table 7).The integrated model showed excellent predictive performance for post-LSG gout risk (AUC = 0.895). At the optimal probability threshold of 0.0822:Sensitivity: 76.0%、Specificity: 90.4%、Youden index: 0.664 (maximum).Individual predictors performed as follows: Male sex (AUC = 0.742, *P* < 0.001) and ΔTBW% (ΔTBW% < 9.96%; AUC = 0.740, *P* < 0.001) demonstrated significant and comparable predictive value, with sensitivities of 76.0% and 80.0% respectively.ΔSUA (ΔSUA% >19.59%; AUC = 0.693, *P* = 0.001) showed moderate predictive power (sensitivity 76.0%, specificity 60.0%).History of gout had limited standalone predictive value (AUC = 0.615, *P* = 0.051) despite high specificity (95.1%), with sensitivity of only 28.0% (Fig. 1).

**Table 7:** ROC curve analysis for postoperative gout flare prediction

Factor	AUC	p-Value	95% CI	Cutoff	Youden Index	Sensitivit（%）	Specificity（%）
Gender	0.742	＜0.001	（0.642，0.841）	0.5*	0.484	76	72.4
History of Gout	0.615	0.051	（0.487，0.744）	0.5*	0.231	28	95.1
ΔTBW%	0.74	＜0.001	（0.639，0.84）	9.96	0.488	80	68.8
ΔSUA%	0.693	0.001	（0.575，0.811）	19.59	0.36	76	60
Integrated Model	0.895	0.001	（0.841，0.95）	0.0822	0.664	76	90.4

*Cutoff 0.5 for binary variables (Male=1, Female=0; Gout History=1, No History=0)*

## Discussion

In this retrospective cohort of 535 patients undergoing LSG, the incidence of HUA significantly increased from 55.9% preoperatively to 65.8% at 1 month, while acute gout attacks occurred in 4.7% of patients.It is important to note that these incidence figures are presented without adjustment for potential confounders such as detailed dietary purine load, diuretic use, or specific perioperative hydration protocols, which may influence SUA levels. These findings align with prior reports of a short-term rebound inSUA following bariatric surgery [13, 14]. For example, Lu et al. [13] documented a postoperative SUA peak of 444.8 µmol/L at 1 month, closely resembling our median postoperative level (487 µmol/L). Similarly, Xu et al. [8] reported that early SUA fluctuations were strongly linked to acute metabolic changes, corroborating the pattern observed in our cohort. Notably, the prevalence of HUA in our cohort was markedly higher than that in the general Chinese population (13.3%) [15], emphasizing the unique vulnerability of obese surgical candidates.

Our multivariate analysis identified male sex, younger age, higher preoperative SUA, lower preoperative CR, and higher postoperative CR as independent risk factors for HUA. Male predominance has been consistently reported in both epidemiological and surgical studies.In our cohort, the risk of postoperative HUA was 7.36-fold higher in men than in women. This difference may be partly explained by sex hormone–mediated regulation of renal urate transport: androgens have been shown to upregulate urate transporter 1 (URAT1), enhancing tubular reabsorption of urate, whereas estrogens suppress urate reabsorption. Consequently, premenopausal women with higher estrogen levels exhibit greater urate excretion capacity and lower SUA levels [16, 17]. These mechanisms are consistent with the sex differences reported by Pang et al. [18]. The effect of preoperative SUA, although modest on a per-unit basis, translates into substantial risk across physiological ranges, consistent with the findings of Menenakos et al. [19], who demonstrated that baseline SUA was a predictor of weight-loss success and metabolic outcomes after LSG. The so-called “creatinine paradox,” observed here, has also been highlighted in prior metabolic studies [17]. However, given the lack of direct muscle mass measurement, our interpretation remains hypothetical and reverse causation cannot be excluded.

For postoperative gout flares, our results highlight male sex, a history of gout, larger reductions in total body water percentage (ΔTBW%), and greater increases in SUA (ΔSUA%) as independent predictors. Similar to Soricelli E et al. [20] and Song K et al. [21], who described frequent gout attacks after bariatric surgery, we observed a 21.9% flare rate among patients with pre-existing gout, underscoring the importance of history as a major risk factor. Our identification of ΔTBW% and ΔSUA% as key predictors adds novel evidence: while previous studies [8, 9] emphasized absolute SUA levels, our findings support the “threshold breach” hypothesis [22], whereby rapid fluctuations rather than absolute concentrations drive crystal precipitation and inflammation. The dissociation between gout history and HUA—where history predicted flares but not HUA—further supports the notion that hyperuricemia is necessary but insufficient for acute attacks, with additional triggers such as pre-existing monosodium urate crystal burden, inflammatory activation, or mechanical mobilization likely involved [23].

Several mechanisms may contribute to the short-term rise in SUA after LSG. Prior clinical and experimental work suggests roles for (i) accelerated catabolism and ketosis, where competition between ketones and urate at renal tubular transporters reduces excretion [24]; (ii) dehydration, common in the immediate postoperative period, leading to increased antidiuretic hormone secretion and urate reabsorption [25, 26]; and (iii) tissue hypoxia, which drives ATP breakdown and uric acid generation [27]. Our findings of postoperative CR elevation and ΔTBW% changes are consistent with these mechanisms, supporting their relevance in the bariatric setting.The potential role of perioperative urate-lowering therapy (ULT) in high-risk patients should be considered. A recent prospective study demonstrated that continued ULT after bariatric surgery in patients with gout was associated with a reduced risk of postoperative gout attacks [28]. This suggests that targeted ULT may be a promising strategy for high-risk individuals in the early postoperative period.

The predictive models established in this study showed good performance (AUC = 0.802 for HUA; AUC = 0.895 for gout), outperforming individual predictors. Previous models for postoperative metabolic complications have generally focused on weight-loss outcomes or glycemic control [29], with limited data addressing urate-related outcomes. Nonetheless, calibration was not performed and external validation was lacking, which limits immediate clinical applicability. In contrast, WOJCIAK et al. [25] in a meta-analysis stressed the long-term urate-lowering effects of bariatric surgery but did not address early postoperative risks, highlighting the novelty of our contribution.

This study has several limitations that should be considered when interpreting the results. First, its retrospective single-center design may introduce selection bias, and the relatively short follow-up period of one month prevents the assessment of longer-term SUA trajectories, which are known to generally decline by 3–6 months after surgery [30]. Second, we were unable to fully adjust for several potential confounding factors, including dietary purine intake, diuretic use, and the potential residual effects of preoperative ULT. Although ULT was discontinued perioperatively, prior treatment may have influenced baseline SUA levels or pre-existing crystal burden, which could affect the observed postoperative SUA fluctuations. Third, body composition was assessed using BIA, which is susceptible to variations in hydration status—a particular concern in the early postoperative period when fluid balance is often dynamic and may not be stable. Finally, the diagnostic criteria for hyperuricemia were based on standard purine dietary conditions, which likely do not reflect the actual dietary patterns of patients during the perioperative phase, potentially affecting the accuracy of HUA incidence estimates.

In conclusion, our findings demonstrate that the immediate postoperative period after LSG is a high-risk stage for HUA and gout. While the identified risk factors and predictive models provide valuable insights for early risk stratification, prospective multicenter studies with mechanistic biomarkers, extended follow-up, and external validation are needed to optimize perioperative management strategies for uric acid control.

## Conclusions

This study demonstrates that the early postoperative period after LSG is a vulnerable stage for HUA and gout flares. Male sex, younger age, higher preoperative SUA, lower preoperative creatinine, and higher postoperative CR were identified as independent predictors of HUA, whereas male sex, gout history, larger decreases in TBW%, and greater increases in SUA were predictors of postoperative gout attacks. These findings highlight the multifactorial nature of urate dysregulation after bariatric surgery, involving catabolic stress, fluid shifts, and renal excretory changes.

Our predictive models showed good discriminative ability for both HUA and gout, but lack of calibration, short follow-up, and absence of external validation limit their immediate clinical utility. Therefore, these models should be regarded as exploratory tools rather than definitive decision aids.

Clinically, our results suggest that male patients and those with a prior history of gout warrant closer monitoring in the immediate postoperative period. Attention to hydration status and perioperative uric acid fluctuations may help mitigate early complications. Future multicenter, prospective studies with longer follow-up and external validation are necessary to refine predictive models and to evaluate targeted preventive strategies for urate control after bariatric surgery.

## Data Availability

The datasets used and/or analysed during the current study are available from the corresponding author on reasonable request.

## Abbreviations

LSG: Laparoscopic Sleeve Gastrectomy
HUA: Hyperuricemia
BMI: Body Mass Index
SBP: Systolic Blood Pressure
DBP: Diastolic Blood Pressure
TBW%: Total Body Water Percentage
FFM: Fat-Free Mass
BFP: Body Fat Percentage
FBG: Fasting Blood Glucose
CR: Creatinine
SUA: Serum Uric Acid
TC: Total Cholesterol
TG: Triglycerides
